# 

**Published:** 1881-01

**Authors:** 


### INDEX.

PAGE.

Abscess of hip ........ 131
Acute congestion of the lumbar region of the spinal cord........ 189
Agriculture, the new commissioner of........ 182
Alligator sunstruck........ 293
American Pork........ 211
Amputation of tongue........ 130
Animal, distance can swim........ 282
pests in Greenwood Cemetery........ 297
Animals, effect of lightning upon........ 203
how to kill with a blow........ 281
Pasteur's experiments in vaccinating........ 286
sanitary care of the lower........ 46
spurious pregnancy in the lower........ 64
standard rations for working........ 151
vaccinating........ 264
Anthrax, experiments regarding........ 43
in Louisiana........ 289
in the west........ 210
malignant pustule and the œdema of........ 66
Aquarium, the New York........ 144
Arrowsmith, W. H.—contagious keratitis in the cow........ 274
Autopsies, notes on........ 190

Bates, Erskine S.—veterinary education........ 230
Beard, Geo. M.—trance and trancoidal states in the lower animals...... 90
Berns, G. H.—enteritis........ 38
Bi-chromate of potassa........ 209
Bill to prevent spread of contagious diseases........ 212
Birds, the faculty of articulate speech in........ 227
Black-leg ........ 142
Blood globules........ 202
in hibernation........ 283
letting in the west........ 195

Boa Constrictor........ 290
Bogus veterinary medical degrees........ 257
Bowley, G. W.—posterior paralysis in mules from entozoa in the kidneys 118
phagedæna, a new disease........ 199
Brackett, E. I.—a case of suppurating epididymitis........ 277
Breeding in its relations to physiology and pathology........ 185
physiological aspects of........ 197
Brown, Arthur Erwin.—sterility of mammalia under confinement........ 28
institution........ 147
Bumblefoot........ 67

Calculus, urethral, in a gelding........ 278
Canine epileptic affection........ 203
teeth, Clarke on the uses of........ 148
Carpenter, W. H.—Epizooty at San Francisco........ 270
Case department........52, 130, 187, 267
Cattle commission, a national........44, 75, 263
treasury........ 260
contagious diseases among American........ 77
disease, another new........ 292
increased transportation of........ 73
the trade in live........ 77
transit of in railway cars........ 74
Chicken cholera, treatment of........280–286
Commissioner of Agriculture, the new........ 182
Colleges, new veterinary........ 259
Concretions in large intestine of a swan........ 191
Congress and veterinary medicine........ 148
Conklin, W. A.—dogs killing swan........ 63
Convulsions, uræmic, in a pug........ 194
Colic, a peculiar........ 289
Columbia veterinary college........149–209
Contagious diseases........ 261
of animals in Germany, laws relating to........ 207
among American cattle........ 77
of animals, State help for investigations in the........ 43
Correspondence........61, 136, 193
Cows, abortion in........ 66
catarrhal phthisis in........ 277
contagious keratitis in........ 274
Cow, death of a famous Jersey........ 290
inversion of the vagina of a........ 213
ration of ensilage for a........ 204

Cows, remaining in heat.... 65
Cow, scirrhous carcinoma in a.... 56
Cressey, Noah—diseased meat and its consequence upon our health and happiness.... 15
Cribbing.... 211
Cuff, John—a case of acute congestion of the kidney urethral calculus in a bay gelding.... 278

D. C. L.—uræmic convulsions in a pug.... 194
Deaths.... 210
Deer, an epidemic among.... 212
Deformity, a curious.... 297
Diarrhœa in calves, treatment of.... 211
Diffuse nephritis, a case of chronic.... 53
Disease, a new.... 70
" in horses' feet, the cause and cure of.... 162
strange cattle.... 145
Diseases of animals in captivity.... 205
appropriations for experiments.... 74
preventing by inoculation....184, 193
unknown, the appearance of.... 261
Diseased meat and its consequence.... 15
Doe, bloody infiltration of pelvic cellular tissue in a.... 191
Doctoring through the newspapers.... 183
Dog distemper, cure for.... 140
home and hospital.... 212
Dogs, hydrophobia among, in New York.... 263
splenic fever in the.... 282
Drill, extensive caseous deposits in the lungs of a.... 191
Dropsy in horses.... 206
Dysentery in dogs.... 210

Editorial department....41, 121, 178, 257
Education, veterinary.... 230
Elephant, a case of menengitis in the.... 61
dentistry at the zoo.... 214
Endometritis, chronic, in a mare.... 249
Ensilage and disease....126, 262
Enteritis.... 38
Epidemic, a curious.... 290
Epididymitis, a case of suppurating.... 277
Epizooty again.... 294
at San Francisco.... 270

Epizootic discharges, contagiousness of ..... 70
Equina, or horse-pox ..... 210
Euphorbia villosa ..... 290
Eyes, a disease of ..... 289

Farcy ..... 205
Fasting frogs ..... 74
  horses ..... 76
Fatty fibroma, removal of ..... 188
Fibrinous encocarditus, cases of ..... 202
Firing horses ..... 279
Flaxseed meal ..... 209
Foals three at a birth ..... 281
Foot and mouth disease ..... 210
  in England ..... 73–147
  in New York City ..... 145
  in the United States ..... 136
  in this country ..... 147
  salicylic acid in ..... 145
Foot rot ..... 140
Fouquet's method of procuring either sex at will ..... 280
Fowl cholera in the west ..... 68
Fowls, roup in ..... 142
  scurfy legs in ..... 67
Frey, Mark L.—removal of fatty fibroma ..... 188

Haas' hog-cholera remedy, analysis of ..... 145
Hammond, William A.—have birds the faculty of articulate speech ..... 227
Heath, Allen S.—the physiological aspects of breeding ..... 197
  sanitary condition New York City stables ..... 36–115
Hemorrhagic spinal meningitis in a monkey ..... 190
Hens, dyspepsia in ..... 141
Hibernation, the blood in ..... 2[illegible]3
Hip, lacerated wound of ..... 1[illegible]8
History of the horse .....
Hog cholera and trichinosis scare ..... 1[illegible]6
  losses from ..... 74
  on Long Island ..... 134
  treatment of ..... 139
Hopkins, James D.—foot and mouth disease in the United States ..... 136
Horse, a giant ..... 70
  a new parasite of the ..... 64
  a case of fibrinous pericardites in the ..... 57

Horse, composition and amount of urine in a.......... 138
a case of superfetation in the.......... 214
a new.......... 281
and the man.......... 294
cystic change in the mucous membrane of the stomach of a...... 131
disease in St. Louis.......... 295
the turcoman.......... 284
feeding, experiments in.......... 65
fever, the.......... 296
hemiplegia following influenza in a.......... 133
history of.......... 79
irido-chorodites in.......... 106
in spectacles.......... 210
instructive article on the.......... 281
paraplegia in the.......... 144
posterior paralysis of the..........170–189
rupture of the stomach.......... 206
rabies in, a case of.......... 202
trade in Cincinnati.......... 77
Horses, an epidemic among..........145–213
chloral as a sedative for.......... 138
dropsy in.......... 206
eye, how to examine the.......... 138
fasting.......... 76
feet, the cause and cure of disease in.......... 162
foot when shod and unshod. expansion of.......... 245
heavy draught breeding.......... 47
malaria in.......... 280
of New York.......... 210
old and new, English race.......... 290
standard rations for..........64–71
shall our, wear shoes.......... 178
total number of, in the world.......... 70
treatment for piles in.......... 138
House, Charles D.—sued by L. C. Bruce.......... 70
Hybridism in ornithology.......... 298
Hydrophobia, a cure for.......... 291
among the dogs in New York.......... 263
Hydrophobic saliva, a new disease developed from.......... 139
Hyena, spinal paralysis in.......... 273

Idoform in ozœna.......... 202
Inoculation, preventing diseases by..........184–193

Investigation of contagious diseases among domestic animals............... 121
Irido-choroiditis in the horse.......................................................... 106

Journal of comparative medicine..........................................48–127

Keratitis contagious, in the cow......................................................... 274
Kidney, a case of acute congestion of the........................................ 55

Lacerated wound of the neck............................................................. 130
hip................................................................. 187
tongue.......................................................... 187
Lampas ............................................................................................ 280
Landy, Louis H.—history of the horse.............................................. 79
Laws relating to contagious diseases of animals in Germany........ ...... 207
Lice, destruction of........................................................................... 211
Lindsay, J.—a case of chronic diffuse nephritis.................................. 53
Liver rot in sheep, dangers of............................................................. 287
Malaria in horses................................................................................ 280

Mammalia, sterility of, under confinement........................................ 28
Manure nuisance in New York........................................................... 292
Mare, chronic endometritis in............................................................ 249
Mares, blind, for breeding................................................................. 71
McLellan, E. A.—the cause and cure of diseases in horses' feet............ 162
Meat, selling diseased....................................................................... 146
Meningitis, hemorrhagic spinal, in a monkey...................................... 190
Milk as a cause of death.................................................................... 292
fever, preventive and treatment of.................................................... 285
sickness ........................................................................................ 69
Miller, Wesley—preventing diseases by inoculation............................ 193
Monkey, hemorrhagic spinal meningitis in a....................................... 190
intestinal concretions in the.......................................................... 281
phthisis in a.................................................................................. 60
singular instance of depraved appetite in a.................................... 193
Monstrosity in fowls........................................................................... 211
Moon blindness, treatment of............................................................. 139
Moore, William O.—irido-choroiditis, commonly called moon-blindness, in a horse.................................................................................. 106
Mule, another fertile.......................................................................... 209
a cross-eyed.................................................................................. 209
Mules, posterior paralysis in.............................................................. 118
Muscular exertion, the effect of, on proteid metabolism...................... 151
Museums for private veterinary practitioners........... .......... ............. 212

Mustang liniment........ 144

National live stock journal........ 72
Navin, John N.—a curious veterinary experience........ 195
the expansion of the horse's foot when shod and unshod........ 245
three days' western experience........ 276
News and miscellany........70, 144, 209, 289
Newspapers, doctoring through the........ 183
New York city stables........36, 114
Nitrate of amyl........ 138
Notes on autopsies........ 190

Œdema glottidis, a case of........ 52
Œsophagus, ulceration of........ 275
Ormsby, H. P.—the effect of muscular exertion on proteid metabolism .151
Ostriches, how, digest........ 283
Ox, Steel on diseases of the........ 209

Pachy-meningitis interna........ 253
Paralysis of the horse........ 170
Parasite, a new cestoid........ 208
in pork, a new........ 280
the fluke........ 209
in flies........ 78
Parkinson, George H.—catarrhal phthisis in a cow........ 277
pachy-meningitis interna........ 253
posterior paralysis of the horse........ 189
Parrots, gout in........ 202
Pasteur's experiments in vaccinating animals........ 286
Pen and plough........ 289
Pet animals and contagious diseases........ 261
Phagedæna........ 293
is it a new disease?........186, 199
Phthisis, catarrhal, in a cow........ 277
Pigs, dyspepsia in........ 69
effects of alcohol on........ 69
Pimples, whelps, eruptions........ 289
Plague, the Siberian........ 289
Pleuro-pneumonia, a new cause for........ 209
exportation of contagious........ 290
extinction of, in Holland........ 72
in England........ 210

Pleuro-pneumonia, in American cattle........ 292
on Long Island........ 72
suppressing........ 76
in the United States, extent of........ 146
Pork, a new poison in........ 139
Porter, W. H.—anæsthetics in veterinary practice........ 12
and Parkinson, Geo. H.—on the lesions and pathology of the so-called posterior paralysis of the horse........ 170
and William Soula—a case of œdema glottis following the epizootic catarrh........ 52
Posterior paralysis of the horse........ 189
in mules........ 118
Poisons, persistent effect of animal........ 203
preservation of anthrax........ 202
Prevention of infectious diseases by inoculation........ 41
Profession, what, shall a young man choose........ 262
Progress of veterinary science........64, 138, 202, 280
Rabies, a case of........ 267
in the horse, a case of........ 202
virus of, in cerebral substance of mad animals........ 284
Recent contributions to the literature of comparative medecine........ 1
Rectum, rupture of........ 283
Register, veterinary medical........185, 264
Removal of fatty fibroma........ 188
Reviews........49, 128, 265
Rheumatism, bi-carbonate of potassium in........ 144
Ricketty eggs........ 67
Rupture of the horse's stomach........ 206
Rural physiology........ 146

Sanitary condition, New York City stables........ 36
Satterthwaite, Thomas E.—recent contributions to the literature of comparative medicine........ 1
Scab, treatment of........ 298
Scirrhous carcinoma in a cow........ 56
Sheep, diseases of........ 141
liver rot in, danger of........ 287
round worms in........ 207
Shoes, shall our horses wear........ 178
Skull, exostosis of the........ 253
Snakes, care of........ 291
Sows eating pigs........ 212
Speech, have birds the faculty of articulate........ 227

Splenic fever in Dutchess county.............................................. 294
leucocythemia, a case of .............................................. 202
Spinal paralysis in hyena and puma.............................................. 273
Strange disease.............................................. 295
String-halt.............................................. 284
Swan, concretions in large intestine of a.............................................. 191
Swine, healthfulness of American.............................................. 213

Tapeworm,.............................................. 300
embryo of the.............................................. 69
in Iceland.............................................. 282
Texas cattle fever.............................................. 73
Thompson, Frank J.—singular instance of depraved appetite in a monkey.............................................. 193
Todd, Charles A.—case of meningitis in the elephant.............................................. 61
spinal paralysis in hyena and puma.. .............................................. 273
Tongue, amputation of.............................................. 130
lacerated wound of.............................................. 187
Trance and trancoidal states in the lower animals.............................................. 90
in the lower animals .............................................. 204
Treasury cattle commission.............................................. 260
Trichinæ in chickens .............................................. 203
Trichinosis and American pork.............................................. 149
in Spain.............................................. 76
treatment of, by glycerine .............................................. 1
Trink, James H.—hemiplegia following influenza in a horse .............................................. 133
Tuberculosis and experiments on murderers.............................................. 45
transmission of .............................................. 141
Turtle, a monster.............................................. 292

Ulceration of the œsophagus in a mule .............................................. 275
Unknown diseases, the appearance of.............................................. 261
Urethal calculus in a bay gelding.............................................. 278
Uterus, enormous distension of.............................................. 249
Vaccinating animals.............................................. 264
Pasteur's experiments in .............................................. 286
Vegetable charcoal .............................................. 202
Veterinarian a, selling diseased cattle... ... .............................................. 295
Veterinary colleges, new.............................................. 259, 289
congress.............................................. 210
education.............................................. 47, 230, 295
experience, a curious.............................................. 195

Veterinary hospital .......... 209
hygiene .......... 115
journal, a new .......... 289
law .......... 146, 211
medical degrees .......... 257
medicine in England .......... 144
medical register .......... 185, 215, 264
publications, new .......... 209, 291
practice, anæsthetics in .......... 12
ethics in .......... 125
regulating .......... 70
school, a new .......... 209
society, a .......... 70
surgeons bill .......... 210
British congress of .......... 144
death of .......... 71, 145
social recognition of .......... 144

Wendt, E. C.—notes on autopsies .......... 190
W. E. C.—phthisis in a monkey .......... 60
Western experience, three days' .......... 276
Wheat, L. E.—cystic change in the mucous membrane of the stomach of a horse .......... 131
ulceration of the œsophagus in a mule .......... 275
and Porter, W. H.—chronic endometritis in a mare .......... 249
Wild beasts, food for .......... 293
Zoölogical garden, a new .......... 144
the Central Park .......... 71

